# A Review of the Potential Benefits of Increasing Vitamin D Status in Mongolian Adults through Food Fortification and Vitamin D Supplementation

**DOI:** 10.3390/nu11102452

**Published:** 2019-10-14

**Authors:** William B. Grant, Barbara J. Boucher

**Affiliations:** 1 Director, Sunlight, Nutrition, and Health Research Center, P.O. Box 641603, San Francisco, CA 94164-1603, USA; 2 The Blizard Institute, Barts & The London School of Medicine & Dentistry, Queen Mary University of London, 4 Newark Street, London E12AT, UK; bboucher@doctors.org.uk

**Keywords:** cancer, cirrhosis, ischemic stroke, lower respiratory tract infections, neoplasms, vitamin D deficiency, vitamin D fortification, vitamin D supplementation, 25-hydroxyvitamin D, pregnancy, preterm birth

## Abstract

Serum 25-hydroxyvitamin D (25(OH)D) concentrations are low in Mongolia, averaging 22 ng/mL in summer and only 8 ng/mL in winter. Mongolians have high incidence and/or prevalence of several diseases linked to low 25(OH)D concentrations, including ischemic heart disease, malignant neoplasms, cirrhosis of the liver, ischemic stroke, lower respiratory tract infections, preterm birth complications, and diabetes mellitus. Fortifying regularly consumed foods such as flour, milk, and edible oils with vitamin D_3_ could raise 25(OH)D concentrations by about 10 ng/mL. However, to achieve 25(OH)D concentrations of 30–40 ng/mL in adults, vitamin D intakes of 1000 to 4000 IU/day would be required, making personal supplement use necessary. On the basis of prospective observational studies and clinical trials of disease incidence or known mortality rates and adverse pregnancy and birth outcomes, raising mean serum 25(OH)D concentrations to 40 ng/mL would likely reduce incidence and mortality rates for those and other diseases, reduce the rate of adverse pregnancy and birth outcomes, and increase mean life expectancy by one year or more.

## 1. Introduction

Mongolia is a country in Central Asia lying between Russia and China. The population of Mongolia is about 3 million, with about 45% living in the capital, Ulaanbaatar, and about 30% being nomadic or seminomadic. Most inhabitants are of Mongol ethnicity. According to the World Health Organization, life expectancy for males in Mongolia in 2018 was 65.7 years, whereas that for females was 74.2 years. The latitude of Ulaanbaatar is 47.9 °N and its average elevation is 1350 m. The average day/night temperature in Ulaanbaatar in January is −16/−26 °C, whereas in July, it is +25/+12 °C. Because of that location, producing vitamin D_3_ in the skin from solar ultraviolet B (UVB) exposure is impossible for 6 months near the end of the year [1,2].

Mongolia is a relatively poor country. The per capita income expressed as purchasing power parity in 2018 was $13,735 (current international $) [3]. Noncommunicable diseases accounted for 43% of deaths and one-third of total health expenditures in 2013, with cardiovascular disease (CVD) costs accounting for 24% of the total [4]. Even though the population has high levels of health coverage (93%), personal health expenses are high and contributed to an 8% increase in the incidence of poverty in 2012 [5]. Thus, an inexpensive approach for reducing the burden of disease in Mongolia would be useful.

The roles of vitamin D in reducing the risk of many diseases and conditions have been the subject of numerous studies in the past two decades. Vitamin D from UVB exposure and oral intake has been found to significantly reduce risk of many types of cancer [6], acute respiratory tract infections [7], adverse pregnancy outcomes [8], all-cause mortality rate [9], and many more diseases and conditions, and there are many reviews on the non-skeletal effects of vitamin D to which the interested reader is directed [10,11,12].

This paper reviews the evidence regarding the potential role of vitamin D in improving the health status and reducing the disease burden of Mongolians. Those improvements could come about through reductions in the risk of, and hopefully the health costs of, the more common diseases in Mongolia. We also aim to make recommendations on how best to improve the vitamin D status of Mongolians at the population level.

## 2. Methods and Materials

This paper is a narrative review, of those papers presenting evidence that vitamin D reduces risk of incidence, progression, or mortality from various health conditions found through searching pubmed.gov and scholar.google.com. Age-standardized mortality rates for selected health outcomes in males and females in Mongolia in 2016 were obtained from the World Health Organization [13]. In addition, information on diet, which also affects health and can be a source of vitamins D_2_ and D_3_, was obtained from the Food and Agriculture Organization of the United Nations [14] for 2013 and represents consumer dietary supply. Spoilage and waste were not accounted for, but that factor generally accounts for about 10–40% of the supply, depending on the type of food [15].

## 3. Results

To understand the more common diseases in Mongolia, mortality rate data for Mongolia in 2016 were obtained from the World Health Organization [13] (Table 1). On the basis of the difference in life expectancy and all-cause mortality rates for males and females, each 62.6 deaths/100,000/year is equivalent to 1 year of life-expectancy loss [13]. Thus, it should be possible to estimate the reductions in mortality rates and the increases in life expectancy that could be expected through increasing vitamin D status at the population level.

A study in Mongolia measured serum 25-hydroxyvitamin D (25(OH)D) concentrations in 160 residents in summer 2011 and in winter 2013 [16], as shown as means in Table 2. Females had concentrations 3–5 ng/mL lower than those of males in summer (Figure 2 in [16]). Vitamin D intake from the Mongolian diet was estimated in that study at 0–40 IU/day in winter and 0–55 IU/day in summer. However, those intake values may be underestimates because they did not include 25(OH)D in animal products, which could double the estimates [17]. Thus, it is not surprising that 40–70% of Mongolian children had signs of rickets in the 1990s [18]. However, in 2010, the estimate of childhood rickets prevalence had dropped to ~20.5% [19].

### 3.1. Cardiovascular Disease

The role of vitamin D in reducing risk of CVD is unresolved. Prospective observational studies show that low 25(OH)D concentrations are associated with greatly increased risk of CVD. A study in Denmark examined the relationship between baseline 25(OH)D concentration and risk of ischemic heart disease (IHD), myocardial infarction (MI), and early death [20] in 10,170 participants (mean age at enrollment ~57 years), initiated in 1976–1978 and followed up until 2001–2003. Multivariate rates of IHD, MI, and early death were all significantly higher for people with seasonally adjusted 25(OH)D concentrations <10 ng/mL vs. >30 ng/mL. Further analysis showed these differences were significant for fatal but not for nonfatal IHD or MI for 25(OH)D < 10 ng/mL; that paper included a meta-analysis for IHD based on 19 prospective observational studies, finding 33% (fixed effects) or 39% (random effects—used when heterogeneity exists between studies [21]) increases in risk for lowest vs. highest quartiles of 25(OH)D concentrations. On the basis of the three studies that accounted for 55% of the size (weight) of the effects of vitamin D status on IHD risk in that meta-analysis, low 25(OH)D was defined as <~16 ng/mL and high as >~30 ng/mL, and similar results were found for early death.

However, a meta-analysis of 21 randomized controlled trials (RCTs) that included 83,291 patients—half of whom received vitamin D supplements—did not show supplementation to reduce the risks of major adverse cardiovascular events, CVD mortality, stroke, or all-cause mortality [22]. VITAL RCT found that supplementing with 2000 IU/day of vitamin D_3_ for a median time of 5.3 years had no effect on CVD incidence [23]. The disagreement between observational studies and RCTs regarding CVD outcomes with recent vitamin D status appear to conflict; however, possible explanations for the discrepancy include that few of the RCT participants in that meta-analysis had 25(OH)D concentrations <16 ng/mL and that the supplementation periods were all shorter than the natural history of the progression of atheromatous disease.

Because many Mongolians of all ages have serum 25(OH)D concentrations <10–15 ng/mL in winter and early spring, raising those concentrations through long-term vitamin D supplementation, at least during those seasons, could well reduce the risk of CVD events.

### 3.2. Stroke

In a meta-analysis of 16 prospective observational studies, researchers found that low vs. high 25(OH)D concentration was associated with a 32% increased risk of ischemic stroke (relative risk (RR) = 1.32; 95% confidence interval (CI), 1.19 to 1.48) [24]. A meta-analysis based on three case–control studies of association found the RR = 6.59 (95% CI, 1.17 to 37.02) [24]. In a case–control study from Brazil, the fully adjusted odds ratio (OR) for ischemic stroke for 25(OH)D <20 ng/mL vs. >30 ng/mL was 17 (95% CI, 6 to 32) [25]. However, stroke patients in that study had twice the rate of diabetes mellitus (DM) of control subjects, five time the rate of smoking, twice the rate of antihypertensive medication usage, and 25% lower 25(OH)D concentrations, suggesting that at least some of those risk factors (e.g., obesity [26], air pollution, and smoking [27]) had probably contributed to the low 25(OH)D concentrations. Vitamin D status does not seem to affect risk of hemorrhagic stroke. A prolonged observational study in Hawaii reported a significant reduction in risk of ischemic but not hemorrhagic stroke with higher vitamin D intakes [28]. Unfortunately, hemorrhagic stroke death rates in Mongolia are 12 times those of ischemic stroke, so increasing 25(OH)D concentrations will not significantly lower Mongolian stroke rates. Another review shows the reducing order of importance of risk factors for stroke for males in Mongolia is smoking, hypertension, insufficient physical activity, obesity, DM, and hypercholesterolemia; and for females is hypertension, insufficient physical activity, obesity, DM, hypercholesterolemia, and smoking [29]. Since smoking rates in Mongolia for 2015 were 40% for males and 4% for females [30], this difference helps explain the much higher stroke rates and probably the higher mortality rates for males than females.

### 3.3. Type 2 Diabetes Mellitus

Mounting evidence from observational studies and clinical trials indicates that vitamin D reduces the risk of type 2 DM (T2DM). One prospective study reported inverse associations between baseline serum 25(OH)D and future glycemia and insulin resistance after 10 years [31]. A meta-analysis of nine observational studies that included 28,258 participants with a mean age of 68 years, followed up for a median time of 7.7 years, reported an RR of 1.31 (95% CI, 1.11 to 1.54) for developing T2DM for low vs. high 25(OH)D concentrations [32] that remained significant after adjustment for potential confounding factors (RR = 1.17 (95% CI, 1.03 to 1.33; *p* = 0.02)). Another meta-analysis of 71 studies reported a significant inverse relationship of vitamin D status with measures of glycemia in both diabetic (*r* = −0.22 (95% CI, −0.18 to −0.26; *p* = 0.000)) and nondiabetic (*r* = −0.07 (95% CI, −0.05 to −0.09; *p* = 0.000)) subjects [33].

Two recent RCTs reported a reduced risk of progressing from prediabetes to T2DM with supplementation; the first, in Iran, involved 162 participants aged 18–80 years with mean 25(OH)D concentration of ~12.5 ng/mL, fasting insulin of ~15 µU/mL, and a homeostatic model assessment of insulin resistance score raised at ~3–4 [34] and either impaired fasting glucose (IFG), impaired glucose tolerance (IGT), or both. The intervention group was given weekly “pearls” of 50,000 IU of vitamin D_3_ for 3 months, followed by 50,000 IU/month for another 3 months. The control group received placebo. The mean serum 25(OH)D concentration in the treatment group rose to 41 ng/mL after 3 months, declining to 36 ng/mL after 6 months, by which time seven in the control group had progressed from IFG to IGT vs. none in the treatment group, whereas four in the control group had progressed from IFT ± IFG to T2DM vs. one in the treatment group. Thus, 11 in the control group had progressed vs. one in the treatment group (*p* = 0.002). The second recent RCT studied 2423 prediabetic participants with a mean 25(OH)D concentration of ~28 ± 10 ng/mL [35], a mean age of 60.0 years, a mean body mass index (BMI) of 32.1 kg/m^2^ of body surface area, and a mean glycated hemoglobin level of 5.9% (48 mmol/mole). A total of 84.2% of the participants met the glycemic criteria for prediabetes for both fasting plasma glucose and glycated hemoglobin, and about one-third of subjects met all three glycemic criteria. The treatment group was given 4000 IU/day of vitamin D_3_, vs. placebo in the controls. By the end of the trial, diabetes had developed in 616 patients (293 in the treatment group vs. 323 in the control group). Although the risk of progression to T2DM for vitamin-D-supplemented subjects compared with that of subjects on placebo was not significantly reduced overall (hazard ratio (HR) = 0.88 (95% CI, 0.75 to 1.04; *p* = 0.12)), it was reduced (as reported in Table 2), significant, or suggestive for several prespecified subgroups. Namely, for those with BMI < 30 the HR was 0.71 (95% CI, 0.53 to 0.95); for non-Hispanics, HR = 0.86 (95% CI, 0.72 to 1.02); for males, HR = 0.82 (95% CI, 0.68 to 1.01); for a median age of >60.9 years, HR = 0.80 (95% CI, 0.64 to 1.01).

A further report of a vitamin D RCT involving prediabetics and recently diagnosed T2DM patients randomized to receive 5000 IU/day of vitamin D_3_ or placebo showed that at 6 months, mean 25(OH)D had reached 51 ± 10 ng/mL and 21 ± 7 ng/mL in the treatment and placebo groups, respectively (*p* < 0.001) with an improved M value, an index of peripheral insulin sensitivity after supplementation (mean change [95% CI]: 0.92 [0.24 to 1.59] vs. −0.03 [−0.73 to 0.67]; *p* = 0.009), and a greater glucose disposition index (mean change [95% CI]: 270 [−340 to 880] vs. −60 [−700 to 590]; *p* = 0.04) after 6 months supplementation, but no other changes in outcome [36].

Thus, long-term improvement in the vitamin D repletion of healthy adults and those with early features of prediabetes (with intakes of ~4000 IU/day of vitamin D_3/day_) would be very likely to reduce the risk of T2DM development in Mongolia.

### 3.4. Hypertension

Hypertension is a growing concern in Ulaanbaatar, possibly due to the high ambient air concentrations of particulate matter with a diameter <2.5 µm (PM_2.5_) [37] with health effects similar to those of smoking—an important risk factor for ischemic stroke [38]. Hypertension is also an important risk factor for stroke, especially ischemic stroke in Mongolia [29] as well as for progressive atherosclerosis (with vascular calcification) in Mongolian women [39]. However, screening for hypertension is not widely available, and thus any reductions in hypertension from the long-term prevention of vitamin D deficiency (which suppresses renin production [40,41]) as has so far only been reported in young men [42], perhaps because progression of atheromatous disease becomes increasingly irreversible, would be useful [43].

According to meta-analyses of RCT data, vitamin D supplementation has modest effects on blood pressure in general, with the largest effects in people with hypertension (decrease of 6.6 mmHg [95% CI, −8.7 to −4.4] for systolic blood pressure and a decrease of 3.1 mmHg [95% CI, −4.7 to −1.5] for diastolic blood pressure) [44], which might be larger with higher dosages. An open-label study conducted in Canada where people were given vitamin D_3_ capsules and counseled to take a daily amount of vitamin D_3_ between 1000 and 20,000 IU/day to achieve 25(OH)D concentrations of >40 ng/mL included a nested case—control group of hypertensive participants aged 56 ± 15 years with a mean baseline 25(OH)D concentration of 35 ng/mL who increased their mean 25(OH)D concentrations to a mean of 45 ng/mL after 1 year and those participants had significant reductions in systolic blood pressure of −16 ± 20 mmHg and in diastolic blood pressure of −12 ± 12 mmHg [45], with no difference in the size of these effects between those taking or not taking hypotensive medication. Achieving vitamin D repletion could prove useful in reducing hypertensive health problems, since hypertension is a world-wide public health problem that is currently the leading risk factor in the global burden of disease. It is the major modifiable risk factor for heart disease, stroke, and kidney failure. Additionally, chronic kidney disease (CKD) is both a common cause of hypertension and can also be a result of uncontrolled hypertension. The combination of hypertension and CKD greatly increases the risk of adverse cardiovascular and cerebrovascular outcomes [46].

### 3.5. Chronic Kidney Diseases

One function of the kidney is to convert 25(OH)D to 1,25-dihydroxivitamin D (1,25(OH)_2_D). Thus, when kidney function is impaired as in CKD, serum 1,25(OH)_2_D concentrations decline, leading to greater withdrawal of calcium from bones to maintain normal serum calcium concentrations and thereby lowering bone mineral density [47]. Thus, itamin D deficiency in CKD leads to renal osteodystrophy with deterioration of bone quality. The risks of non-skeletal disorders such as metabolic syndrome, hypertension, immune dysfunction, hyperlipidemia, diabetes, and anemia also increase, likely because non-bony tissues activate vitamin D to form calcitriol and express the vitamin D receptor, allowing vitamin D to have many biological roles other than those relevant to bone health [48].

Pharmacological treatment with 1,25(OH)_2_D or its analogs with the aim of protecting bone from renal osteodystrophy often causes hypercalcemia and hyperphosphatemia without major benefits to bone health, and can increase vascular calcification [49]. Conversely, intact vitamin D_3_ supplementation reduces those risks and is thought to have a role in managing bone and cardiorenal health and to reduce the abnormally increased mortality of patients with chronic renal disease [49]. Thus, a combination of intact vitamin D and hormonally activated vitamin D or its analogs is thought to be the most beneficial for use in renal failure, not least because local 1α-hydroxylase activity allows vitamin D activation in relevant tissues, including the parathyroid glands [49]. This increases serum calcitriol concentrations and reduces the risks of secondary complications such as hypertension, muscle weakness, falls, and increased cardiovascular risks and mortality rates [47]. Clinical trials of vitamin D in CKD more often use analogs of 1,25(OH)_2_D than intact vitamin D_3_, but whether they reduce mortality rates in CKD is not apparent [50].

A prospective 3-year study of 470 unsupplemented non-dialysis patients in Spain with stage 3–5 CKD reported that those with baseline 25(OH)D values between 20 and 30 ng/mL had better survival, lower hospitalization, and better composite renal endpoints than those with lower or higher baseline concentrations [51], but found no additional benefits from raising serum 25(OH)D concentration to >30 ng/mL.

### 3.6. Chronic Obstructive Pulmonary Disease

Chronic obstructive pulmonary disease (COPD) is an umbrella term describing progressive lung disease previously called emphysema, chronic bronchitis, or refractory (nonreversible) asthma, and is characterized by increasing breathlessness. (see the website: https://www.copdfoundation.org/).

A meta-analysis of data from 21 studies covering 4818 patients and 7175 control subjects reported lower serum 25(OH)D concentrations in COPD patients than in controls (standardized mean difference (SMD), −0.69 (95% CI, −1.00 to −0.38; *p* < 0.001)), which was most marked in severe COPD (SMD, −0.87 (95% CI, −1.51 to −0.22; *p* = 0.001)) and in COPD exacerbations (SMD, −0.43 (95% CI, −0.70 to −0.15; *p* = 0.002)), that is, vitamin D deficiency was associated with increased risk of COPD (OR = 1.77 (95% CI, 1.18 to 2.64; *p* = 0.006)), with COPD severity (OR = 2.83 (95% CI, 2.00 to 4.00; *p* < 0.001)) but not with COPD exacerbations (OR = 1.17 (95% CI, 0.86 to 1.59; *p* = 0.33)), though the heterogeneity of the 25(OH)D assays used affected the associations between vitamin D deficiency and COPD risk [52].

A more recent meta-analysis of four RCTs using individual participant data reported that supplementation with 1200–4000 IU/day of vitamin D_3_ reduced moderate and severe exacerbation rates in COPD in patients with baseline serum 25(OH)D concentrations <10 ng/mL (adjusted incidence rate ratio = 0.55 [95% CI, 0.26 to 0.84]) but not in subjects with higher baseline concentrations [53].

The benefits of vitamin D relevant to COPD risk and severity include increases in the production of the antimicrobial peptide cathelicidin (LL-37) and downregulation of proinflammatory cytokines and effects on airway remodeling, together with inhibition of the production of several proinflammatory cytokines and suppression of the T-cell helper Th1 and Th17 responses thought to aggravate the pathogenesis of COPD. Because vitamin D insufficiency also increases the risks of chronic respiratory infections and acute or chronic airway colonization, maintaining vitamin D status (i.e., circulating 25(OH)D concentrations) within an optimal range in patients with COPD should reduce bacterial loading and hence reduce infective exacerbations. Vitamin D repletion is also protective against other disorders commonly seen in COPD, such as osteoporosis, muscle weakness, and cardiovascular disorders [54].

A recent mechanistic study in mice suggested that activated vitamin D reduced lung damage induced by particulate air pollution and promoted pulmonary tissue repair by downregulating the transforming growth factor β1 signaling pathway and modulating local MMP9 expression, facilitating tissue remodeling. Vitamin D repletion also regulated autophagy signaling through Nrf2 transcription factor upregulation, promoting further protection through increases in Nrf2 molecule stability [55].

### 3.7. Acute Respiratory Tract Infections

Acute respiratory tract infections (ARTIs) occur more often in winter, prompting the hypothesis that solar UVB exposure and increased vitamin D production reduce influenza risks [56]. Vitamin D RCTs have supported that hypothesis [57,58]. RCT conducted in Mongolia reported that children with mean baseline 25(OH)D concentrations of 7.5 ng/mL of vitamin D_3_ who were given 300 IU/day vitamin D showed a 50% reduction in ARTIs [59].

A meta-analysis of 25 RCTs involving 10,933 participants and using individual participant data for baseline vitamin D status reported that vitamin D supplementation reduced the overall risk of ARTIs by 12% (adjusted OR = 0.88 [95% CI, 0.81 to 0.96]) [60] overall, but in those receiving daily or weekly vitamin D doses (but not bolus doses) the risk was reduced by 19% (adjusted OR = 0.81 [95% CI, 0.72 to 0.91]), while participants with baseline 25(OH)D concentrations <10 ng/mL had a 70% reduction in risk (adjusted OR = 0.30 [95% CI, 0.17 to 0.53]) compared to the risk seen in those with higher (i.e., >10 ng/mL) baseline concentrations (adjusted OR = 0.75 [95% CI, 0.60 to 0.95]). This ARTI risk reduction reflects the induction of increased secretion of the bactericidal/viricidal compound cathelicidin (LL-37) and of the defensins in humans. An analysis of data on the 1918 pandemic influenza outbreak in the USA showed that communities with exposure to higher annual solar UVB dosages had lower case-fatality rates than those exposed to lower UVB doses [61]. Because the primary cause of death in that pandemic was from secondary infections causing pneumonia, it was hypothesized that both the antimicrobial properties of the increased secretion of LL-37 and the reduction of the cytokine storm seen in severe influenza protected the alveolar lining from the severity of lung damage that would otherwise have allowed secondary bacterial infection to develop into pneumonia.

### 3.8. Tuberculosis

Growing evidence suggests that vitamin D status affects the risk and progression of tuberculosis (TB) [62]. However, an RCT in Mongolia reported that high-dose vitamin D supplementation did not affect “time to sputum culture conversion” for TB patients [63], and a cross-sectional study of Mongolian schoolchildren found no effect of vitamin D status on the risk of TB [64], despite suggestions from studies in white patients that supplementation may reduce TB sputum conversion time in those on anti-TB drugs.

### 3.9. Cancer

Table 3 presents the incidence rates for the more common types of cancer in Mongolia for 2008–2012.

A large body of peer-reviewed literature on ecological studies reports that greater solar UVB exposure and higher vitamin D intakes/status are associated with reduced risks of cancer incidence and mortality rates [6]. Geographic ecological studies in several midlatitude countries have also reported strong inverse correlations between solar UVB doses and incidence and/or mortality rates for up to 20 cancers [6]. The only mechanism with strong support linking UVB exposure with reduced cancer risk is the rate of UV-induced vitamin D production. Vitamin D reduces cancer risks and cancer mortality through known effects on cells, angiogenesis adjacent to tumor tissue, and on metastasis [6,66]. Prospective studies have found that serum 25(OH)D concentrations are inversely correlated with the specific incidence rates for breast [67,68] and colorectal cancer [69]. For breast cancer, 25(OH)D >60 ng/mL vs. <20 ng/mL was associated with a reduction of 80% (HR = 0.20 (95% CI, 0.05 to 0.82; *p* = 0.03)) [68]. For each 10 ng/mL increment in circulating 25(OH)D, colorectal cancer risk was 19% lower in women (RR = 0.81 [95% CI, 0.75 to 0.87]) and 7% lower in men (RR = 0.93 [95% CI, 0.86 to 1.00]) [69]. A recent meta-analysis of 10 RCTs involving 45,197 participants reported that vitamin D_3_ supplementation with variable doses and trial durations led to a 15% reduction in cancer mortality rates (risk ratio = 0.85 [95% CI, 0.75 to 0.96]) [70].

Esophageal and gastric cancer mortality rates are high in Mongolia. Ecological studies in six midlatitude countries (Australia, China, Japan, Nordic countries, Spain, and the USA) report inverse correlations between solar UVB doses and incidence and/or mortality rates for both esophageal and gastric cancer, as did a recent ecological study in the UK [71]. However, in Australia, the reduced risk of esophageal cancer with lifetime ambient UV radiation was limited to esophageal and esophagogastric junction adenocarcinomas, with no reduction for esophageal squamous cell carcinoma [72]. A further review in 2017 reported accumulating evidence from gastric cancer cells, animal models, and clinical trials to suggest that vitamin D deficiency may increase the risk and mortality of gastric cancer, implying that vitamin D supplementation might be a safe and economical way to reduce gastric cancer risks both prospectively and during treatment [73]. Supporting evidence that vitamin D has the potential to reduce the risk of gastric cancer is provided by studies reporting that vitamin D repletion can eradicate *Helicobacter pylori* infection, itself an important risk factor for gastric cancer [74,75].

Good evidence indicates that greater UVB exposure and better vitamin D status reduce primary liver cancer (hepatocellular carcinoma) risks. A nested case–control study from Europe involving 520,000 participants, 138 of whom developed primary liver cancer, reported that higher 25(OH)D concentrations were associated with a 49% (*p* = 0.04) reduction in hepatoma risk [76]. A study from a cohort of male smokers in Finland reported that baseline 25(OH)D concentration <10 ng/mL vs. >20 ng/mL was associated with a 90% increased risk of hepatoma during a 25-year follow-up [77]. Another large nested case–control study in Japan reported a significant inverse trend between serum 25(OH)D concentration and the incidence of hepatoma (*p*_trend_ = 0.006) [78].

Two recent pooled observational studies strongly support a role of vitamin D in reducing cancer risk. The pooling was comprised of participants in two different vitamin D RCTs with those in the GrassrootsHealth.net cohort of people voluntarily taking vitamin D supplements and having serum 25(OH)D concentrations measured by blood spit tests every 6 months by mail. In the first paper, women achieving a serum 25(OH)D concentration >40 ng/mL had a 65% lower all-cancer incidence rate than that of women achieving values <20 ng/mL after a median follow-up of 3.9 years [79]. In the second study, women achieving >60 ng/mL (*p* = 0.02) over a median follow-up period of 4.0 years had an 84% lower rate of incident breast cancer than did those achieving concentrations <20 ng/mL [68].

The results of VITAL, a large vitamin D RCT over a median time of 5.3 years, were released recently [23]. Although all-cancer incidence was not significantly reduced for the 12,500 people treated with 2000 IU/day of vitamin D_3_, the reduction was significant for those with a BMI < 25 (HR = 0.76 [95% CI, 0.63 to 0.90]) and for black subjects (HR = 0.77 [95% CI, 0.59 to 1.01]). Furthermore, cancer mortality rates were significantly lower by ~25% in the supplemented group when data for the first 2 years of the trial were omitted. A recent letter to the editor has pointed out that such secondary analyses should be considered to be useful for well-defined subgroups in large-scale vitamin D RCTs [80].

### 3.10. Cirrhosis of the Liver

The seroprevalence of hepatitis B virus (HBV) and HCV in the general population in Mongolia is high (11.8% and 15%, respectively [81]). Modest evidence also exists to indicate that vitamin D supplementation in combination with Peg-interferon α injection and oral ribavirin improves sustained virus clearance for HCV genotype 1 but not genotypes 2–4 [82,83]. A study in Spain involving mostly HCV genotype 1 patients reported a higher rate of rapid viral response (23.5% vs. 16.1%; *p* = 0.005) and in the reduction of viral load to ≤15 (51.0% vs. 38.6%; *p* ≤ 0.001) for patients starting treatment with peginterferon–ribavirin in the sunnier half of the year than in the darker half of the year [84], suggesting that maintaining vitamin D repletion could prove to be a useful adjunctive measure in treating HCV. 

A meta-analysis of eight studies published between March 2013 and January 2019 involved 1339 patients with liver cirrhosis and mean 25(OH)D concentrations between 7 and 15 ng/mL. Having a 25(OH)D concentration <10 ng/mL was associated with an increased risk of mortality in patients with liver cirrhosis (risk ratio (RR) = 1.79 (95% CI, 1.44 to 2.22; *p* <  0.01)) [85] and adjustment for publication bias reduced the RR, though it remained significant at 1.62 (95% CI, 1.32 to 1.99; *p* < 0.01).

### 3.11. Alzheimer’s Disease

The primary modifiable risk factor for Alzheimer’s disease (AD) appears to be diet. A typical study reported that for reduced risk of AD, “the identified nutrient combination was associated with higher intake of vegetables, fruit, whole grains, fish and legumes, and lower intake of high-fat dairies, meat and sweets” [86]. An ecological study reported that countries with the highest meat consumption (Brazil, Mongolia, and the U.S.) had the highest rates of AD [87] (the data for Mongolian AD rates used came from people living in Inner Mongolia [88]). However, increased vitamin D intakes/status appears to reduce the risk of AD [89]. Mutton appears to be the preferred meat in Mongolia. Unfortunately, although some meats supply reasonable amounts of vitamin D, both as intact vitamin D_3_ and as 25(OH)D, mutton does not [90], probably because sheep have thick wool coats that greatly reduce the amount of UVB reaching their skin.

Another plausible reason for high AD rates in Ulaanbaatar is the high air pollution rates. A study in Mexico City, at a similar elevation (2300 m vs. 1350 m for Ulaanbaatar) reported that for healthy, normal-weight 11 ± 3 year-olds, exposure to PM_2.5_ concentrations near 20 µg/m^3^, together with elevated ozone levels, are “associated with 12 h fasting hyperleptinemia, altered appetite-regulating peptides, vitamin D deficiency, and increases in ET-1 in clinically healthy children. These changes could signal the future trajectory of urban children towards the development of insulin resistance, obesity, type II diabetes, premature cardiovascular disease, addiction-like behavior, cognitive impairment and Alzheimer’s disease” [91].

The Study of Underlying Genetic Determinants of serum 25(OH)D values and Highly Related Traits (SUNLIGHT) Consortium identified four single-nucleotide polymorphisms (SNPs) significant for serum 25(OH)D, accounting for 2.44% of the variance in 25(OH)D in the Canadian Osteoporosis Study and mapping to genes in the vitamin D metabolic pathway, while Mendelian randomization analyses have suggested that a 1-standard-deviation reduction in natural log-transformed 25(OH)D levels increased AD risk by 25% (OR = 1.25 (95% CI, 1.03 to 1.51; *p* = 0.02)) [92].

A prospective study involving 916 participants from the Three-City Bordeaux, France cohort (aged 65+ years, nondemented at baseline) reported a relationship of baseline vitamin D status with cognitive decline and the incidence of AD. In multivariate analysis, participants with 25(OH)D deficiency (*n* = 218) exhibited faster cognitive decline than individuals with 25(OH)D sufficiency over 12 years of follow-up. A total of 177 incident cases of dementia developed (124 diagnosed as AD), and 25(OH)D deficiency was associated with a marked increase in risk of AD (HR = 2.85 [95% CI, 1.37 to 5.97]) [89].

A recent review reported that observational studies have suggested vitamin D deficiency as a risk factor for AD, Parkinson’s disease, vascular dementia, and multiple sclerosis (and other neurological disorders), through variations in vitamin D axis genes and in the actions of vitamin D, whereas ex-vivo studies show that vitamin D activity can both reduce intracerebral amyloid production and increase its clearance from brain tissue in AD. In addition, recent vitamin D intervention studies have reported significant improvement in cognitive performance in subjects with mild cognitive impairment, senile dementia, and AD [93].

### 3.12. Adverse Pregnancy and Birth Outcomes

Several reports of adverse pregnancy and birth outcomes in Mongolia appear in the literature. The classic outcome of vitamin D deficiency during pregnancy and early life is the development of rickets. A study by the Nutrition Research Center, Public Health Institute, Ministry of Health, Mongolia (a survey of 400 households in Mongolia in 2010 involving 524 children aged 2 to 60 months) found that approximately 10–15% had classic signs and symptoms of rickets [19] and that 57% of the children with 25(OH)D concentration <10 ng/mL had one to eight classic signs of rickets. Additionally, at the time of the survey, 52% of women of reproductive age had a serum 25(OH)D concentration <10 ng/mL; none of the women were taking vitamin D supplements. Another indication of vitamin D deficiency among pregnant women in Mongolia comes from a study of the monthly rates of spontaneous abortions [94], which were higher from December to February (60 to 70 per month) than from May to July (25 per month). The authors also found significant correlations of spontaneous abortion rates with air pollution levels of SO_2_, NO_2_, CO, PM_10_, or PM_2.5_. Another recent paper reported that black carbon particles from air pollution can reach the placenta and could cause damage to the developing fetus [95]. One of the direct effects of such pollution is increased inflammation, which vitamin D can reduce [96]. A study in China compared serum 25(OH)D and CYP27B1 concentrations between women with successful pregnancies and women with pregnancy loss and found both inversely correlated with pregnancy loss [97] (CYP27B1 is the enzyme catalyzing further 1-α-hydroxylation of 25(OH)D to form active hormonal 1,25(OH)_2_D). The physiology, pathophysiology, and clinical outcomes related to certain vitamin D axis variations during pregnancy were reviewed recently [98]. In the comparison between women with normal pregnancies and those with pregnancy loss at 7–9 weeks of gestation, 25(OH)D concentrations were 49 and 34 ng/mL and serum CYP27B1 concentrations were 82 and 38 pg/mL, respectively. In comparison with nonpregnant childbearing women, serum 25(OH)D concentrations were 40 and 12 ng/mL and CYP27B1 concentrations were 39 and 12 pg/mL, respectively. The difference in outcomes between the two groups of pregnant women can be explained by the difference in vitamin D status [99,100]. Thus, the finding in Mongolia is much more likely to be related to 25(OH)D concentrations than to air pollution levels.

Pregnant women in Mongolia were found to have preeclampsia rates of about 4% (4.4% in Ulaanbaatar and 3.5% in rural regions) [101]—an important risk factor for preterm birth and low birth weight [102]. A meta-analysis involving 21 studies reported that low vs. high maternal 25(OH)D concentration was associated with an OR of 1.62 (95% CI, 1.36 to 1.94) for preeclampsia [75], the OR for the Asian sub-population being 2.07 (95% CI, 1.51 to 2.85). In addition, serum 25(OH)D concentrations in the second and third trimesters of pregnancy were more important risk predictors for preeclampsia than those in the first trimester (OR for second/third trimester = 1.93 [95% CI, 1.43 to 2.60]).

In the period 2010–2011, the rate of preterm birth in Mongolia was found to be 4.6%, excluding stillbirths (4.9% when stillbirths were included) [103]. Higher 25(OH)D concentrations can greatly reduce the risk of preterm birth. In an open-label study conducted in South Carolina in which pregnant women of black, white, Hispanic, and Asian ethnicity were enrolled at their first prenatal visit [104], serum 25(OH)D concentration was measured, the women were given a bottle of 5000-IU vitamin D_3_ capsules, and the women were counseled on a supplementation schedule for achieving values >40 ng/mL. Twenty-five women who achieved concentrations of >40 ng/mL had a 60% lower (adjusted OR = 0.41 [95% CI, 0.24 to 0.72]) risk of preterm delivery than that of the 49 women who achieved only <20 ng/mL, with no adverse effects from the 5000 IU/day of vitamin D_3_ given.

Vitamin D status has also been found to affect the risk of gestational diabetes (GD). A multiracial longitudinal observational study in the USA reported a serum 25(OH)D concentration <20 ng/mL as the threshold for GD risk, and that lower concentrations in both the first and third trimesters of pregnancy were significantly associated with increased GD risk [105]. A study in Iran reported that women with GD who took a combination of magnesium, zinc, calcium, and vitamin D (at 100 mg, 4 mg, 400 mg, and 200 IU/day, respectively) had significant reductions in serum high-sensitivity C-reactive protein and plasma malondialdehyde, and significant increases in total antioxidant capacity levels in comparison with placebo [106].

A vitamin D supplementation study of pregnant women conducted in Selenge Aimag province in northern Mongolia [107] enrolled groups of ~120 women at 12–16 weeks of pregnancy and assigned to take 600, 2000, or 4000 IU/day vitamin D_3_, with no reported adverse effects. Mean baseline 25(OH)D concentration was ~8 ng/mL and mean BMI was ~26. Mean serum 25(OH)D concentrations at 36 to 40 weeks of pregnancy were 15, 23, and 27 ng/mL for the groups taking 600, 2000, or 4000 IU/day vitamin D_3_, respectively, suggesting that pregnant women in Mongolia would need to take more than 4000 IU/day vitamin D_3_ in order to achieve the serum 25(OH)D values of at least 40 ng/mL 25(OH)D that had been found necessary for significantly reducing the risk of preterm delivery [104].

### 3.13. Oral Health

The most important ways to reduce the risk of dental caries are adequate tooth brushing and avoidance of sugar-laden foods and beverages, though this is never easy. Higher vitamin D is associated with reduced risk of dental caries, as has been known since 1928 when May Mellanby confirmed the causality of that finding in an RCT in boys [108]. The history of the study of UVB exposure in reducing the risk of dental caries and tooth loss was reviewed in 2011 [109]. Ecological studies reported that people living in areas with higher vs. lower solar UVB doses in the U.S. had fewer dental caries. The role of human cathelicidin (LL-37—a polypeptide with antimicrobial and antiendotoxin activities) in reducing the risk of dental caries was known in 2004 [110], but the role of vitamin D in inducing the production of LL-37 was only identified in 2006 [111]. A 2012 paper reviewed the vitamin D clinical trials conducted in the 1930s and 1940s and found that modest doses of vitamin D halved the incidence of dental caries [112]. A study in Sweden reported that in healthy children at 8 years of age, serum 25(OH)D concentration was inversely correlated with numbers of dental caries, with 20 ng/mL being the approximate threshold for benefit [113].

Modest evidence also indicates that vitamin D reduces periodontal disease risks [114,115]. One review concluded that “vitamin D plays a significant role in maintaining healthy periodontal and jawbone tissues, alleviating inflammation processes, stimulating post-operative healing of periodontal issues and the recovery of clinical parameters” [116]. Reduced risks of severe periodontitis were seen with intakes within recommendations for calcium (OR = 0.76 [95% CI, 0.58 to 0.99]), whey ≥ 9.6 g/day (OR = 0.75 [95% CI, 0.58 to 0.97]), and casein ≥32 g/day (OR = 0.75 [95% CI, 0.58 to 0.97]) after adjustment for age, sex, education, smoking, sucrose intake, alcohol consumption, number of teeth, daily brushing, regular visits to the dentist, and chronic illness, regardless of levels of vitamin D intake; and vitamin D intakes were not associated independently with severity of periodontitis [117].

### 3.14. All-Cause Mortality Rate

Because vitamin D affects the risk of many diseases, one might reasonably expect that higher 25(OH)D concentrations would be associated with reductions in later mortality rates; this is indeed the case. A meta-analysis of 32 observational studies reported a linear decrease in the 7-year mortality rate of approximately 50% ± 15% as baseline serum 25(OH)D concentrations increased from <10 ng/mL to 30–39 ng/mL [9]. No further change was evident at concentrations above 36 ng/mL. A Mendelian randomization study reported that variations of SNPs that reduce serum 25(OH)D concentration accounted for a 20% increase in all-cause mortality rate per each 8 ng/mL decrease in serum 25(OH)D [118]. A prospective study of Chinese adults aged >80 years (median age, 93 years) with mean baseline 25(OH)D of 14 ng/mL followed up for 5466 person-years reported that participants with the highest 25(OH)D concentrations had a 39% reduced mortality rate [119]. In support of those data, a 20-year follow-up study in Sweden reported that sun avoidance was associated with a reduced life expectancy of 0.6 to 2.1 years compared with the highest sun exposure [120].

Estimated changes in mortality rates can therefore be calculated for increasing mean 25(OH)D concentrations up to 40 ng/mL from an assumed annual mean 25(OH)D concentration of 15 ng/mL at present (Table 4). The percentage-point reduction is assumed to be about half of what has been reported in observational studies, and near to the reduction reported in meta-analyses. The reductions in estimated mortality rates were rounded to the nearest 5 deaths/100,000/year if the value was >10 deaths/100,000/year, and were found to be between 125 and 250 deaths/100,000/year for males and between 76 and 140 deaths/100,000/year for females. On the basis of data for death rates from a dozen countries from the World Health Organization for 2015 [13] and the reported life expectancies for those countries in 2015, 1 year of life expectancy change would be expected for each 78 deaths/100,000/year for males and for each 59 deaths/100,000/year for females. This finding implies that raising the current mean 25(OH)D concentrations in Mongolia to 40 ng/mL could increase life expectancy by 1.6 ± 0.8 to 3.2 ± 1.6 years for males and to 1.3 ± 0.7 to 2.4 ± 1.2 years for females. Those increases are similar to those estimated previously for the world’s major continents [121] and for Canada [122].

Several umbrella reviews of findings from observational studies exist regarding the risk of adverse health outcomes as a function of serum 25(OH)D concentration [123,124], and Table 5 summarizes estimates of 25(OH)D concentrations found to be associated with optimal reduction of various adverse health outcomes, as reported in the literature. Because vitamin D works through different mechanisms for different diseases—generally through affecting gene expression [125] but often through rapid nongenomic increases in intracellular calcium concentrations [126]—it is not surprising that the estimated optimal concentrations vary for different outcomes.

### 3.15. Air Pollution

Air pollution levels are high in Ulaanbaatar, especially in winter, and monthly mean PM_2.5_ concentrations reached values of 115 to 150 µg/m^3^ from November 2009 to February 2010, with average annual concentrations ranging from 57 to 96 µg/m^3^ depending on location [131]. Pope and colleagues compared PM_2.5_ concentrations and the numbers of cigarettes smoked per day in terms of risk for lung cancer and cardiovascular disease, cardiopulmonary disease, and IHD [132], concluding that each increase in PM_2.5_ concentration of 240 µg/m^3^ was equivalent to smoking 18–22 cigarettes/day. On the basis of mortality rate data from other studies, those authors estimated that pollution could account for ~40% of lung cancer deaths and ~29% of cardiopulmonary deaths [131]. A more recent paper estimated annual average PM_2.5_ concentrations in Ulaanbaatar to be 59 µg/m^3^ [133], and air pollution was calculated using an integrated exposure–response relationship [134] “to be responsible for 33% (lower: 23%, upper: 42%) of all acute lower respiratory tract infection (ALRI) deaths in children, 19% (lower: 9%, upper: 28%) of all chronic obstructive pulmonary disease (COPD) deaths, 27% (lower: 19%, upper: 42%) of all IHD deaths, 24% (lower: 8%, upper: 34%) of all lung cancer deaths, and 42% (lower: 14%, upper: 54%) of all stroke deaths, for a total of 1400 attributable deaths (lower: 710, upper: 1900) and 40,000 attributable disability-adjusted life years (DALYs) (lower: 22,000, upper: 55,000)” [133].

Little information is available in the literature regarding vitamin D’s role in protecting the body from the adverse effects of air pollution. However, a considerable body of literature exists on the adverse effects of smoking [135] and on vitamin D’s role in reducing the risk of adverse health effects from smoking such as inflammation [136] and GD in pregnant women [137].

## 4. Discussion

Although RCTs and meta-analyses of observational studies are generally considered the best evidence to use in health studies, vitamin D RCTs and observational studies related to serum 25(OH)D concentrations have some fundamental problems: vitamin D RCTs are commonly based on vitamin D dose, independent of baseline or achieved 25(OH)D concentrations, whereas health outcomes are related to serum 25(OH)D concentrations and not to vitamin D dose. Furthermore, vitamin D doses have often been too low to improve vitamin D status enough to induce biological effects, or baseline 25(OH)D concentrations have been too high for any health benefits to be expected from supplementation [138,139]. Thus, vitamin D RCTs should be based on 25(OH)D concentrations, both baseline and achieved [139]. As for prospective observational studies based on 25(OH)D concentrations, the longer the follow-up time, the lower the predictive value of baseline 25(OH)D appears to be [140,141]. In view of those problems, the papers we cite tend to be those providing the best data for baseline D status, that is, those that allow assessments of potential benefits of vitamin D supplementation in subjects with vitamin D deficiency and those that may also allow assessing the benefits of achieving higher rather than lower degrees of vitamin D repletion. One limitation of observational studies is the possibility of “reverse causation ”, that is, that the disease state has caused the lower 25(OH)D concentrations; such effects would be most likely with deteriorating health confining subjects to spend more time indoors after the initial baseline blood draw for assessing serum 25(OH)D concentration. This is likely in serious, debilitating, and terminal illness, but is otherwise unlikely, and though this concern is often raised, it is seldom verified.

### 4.1. Diet

Diet is one of the main determinants of health. Dietary supply data for Mongolia were obtained from the Food and Agriculture Organization for 2013 [14] and are summarized in Table 6. Generally, about 70% of the supply is said to be consumed. Grains provide the highest caloric intake, followed by meat, milk, sugar/sweeteners, and potatoes/other roots. Meat, animal fat, fish, and eggs are important sources of vitamin D, both as vitamin D and as 25(OH)D [142,143]. However, as already mentioned, mutton is not as good a source as beef or pork. Overall, therefore, Mongolians obtain little vitamin D from food.

### 4.2. Recommendations

On the basis of the foregoing information, one can predict that the health and well-being of many Mongolians would be significantly improved if population-level serum 25(OH)D concentrations were increased, especially in non-summer months. Vitamin D fortification of basic foods such as flour and edible oils could contribute to such a program, as discussed below. However, to achieve 25(OH)D concentrations of 30–40 ng/mL in adults, vitamin D intakes of 1000 to 4000 IU/day would be required, making personal supplement use necessary, at least in the winter months [144,145]. People who are overweight or obese require more vitamin D than others due to storage of intact vitamin D in adipose tissue [146] and volumetric dilution [147]. Both the United States Institute of Medicine [148] and the Endocrine Society [149] have stated that 4000 IU/day vitamin D is safe. Few adverse effects are likely from taking such higher doses because they are below the 10,000 to 20,000 IU/day that can be made in skin from solar UVB exposure [150]. In addition, concern about 25(OH)D concentrations over 60–80 ng/mL seems to be based on observational studies in which some participants had only recently begun supplementing with vitamin D and were therefore placed in the wrong 25(OH)D quartile [151].

A recent review outlined the rationale and provided a plan for achieving adequate vitamin D food fortification. It recommended aiming for intakes of 10 to 20 μg (400–800 IU) for the population to achieve serum 25(OH)D concentrations of at least 20 ng/mL in European populations [152]. Among the evidence presented was that Finland introduced food fortification in fluid milk and fat spreads in 2003 and that doubling the amounts added in 2010 virtually abolished vitamin D deficiency. As a result of that voluntary measure by manufacturers, plus a trend for increased vitamin D self-supplementation, mean serum 25(OH)D concentrations rose from 19 ng/mL in 2000 to 26 ng/mL in 2011 [153], with a rise in the prevalence of vitamin D supplement users from 11% to 41%. Two recent papers presented guidelines for food fortification and/or targeted vitamin D supplementation policies that can be implemented to reduce the burden of conditions related to vitamin D deficiency in vulnerable populations in low- and middle-income countries [154,155]. However, a vitamin D dosing study conducted by Robert Heaney found that supplementation at 800 IU/day would raise serum 25(OH)D concentration by only 7 ng/mL in adults [144], and it would be difficult to fortify food in Mongolia to provide intakes of 800 IU/day and, given the low 25(OH)D concentrations seen in winter, this would not result in most people reaching 20 ng/mL.

During the preparation of this paper, a cost–benefit analysis was published for vitamin D fortification of wheat flour, plus elective vitamin D supplementation for reducing vitamin D deficiency in England and Wales [156]. That analysis recommended fortifying wheat flour at 400 IU of vitamin D_3_ per 100 g with an additional role for supplementing with 400 IU/day for the general population and at 800 IU/day for the elderly, on the basis of the recommendations for reducing falls and fragility fractures. In the UK population, 20% of adults and 16% of children aged 11–18 years are estimated to be vitamin D deficient [157] and the analysis indicated that fortifying wheat flour would reduce the prevalence of vitamin D deficiency by 25% and that by adding selective vitamin D supplementation reduction would reach 33%. An analysis published in 2017 modeled the result of fortifying food to the point where everyone would receive an additional 160 IU/day of vitamin D [158], estimating that for Asian adults in the UK, with current winter and summer 25(OH)D concentrations of 15 and 25 ng/mL, respectively, that measure would increase each of those values by 10 ng/mL.

For a country such as Mongolia, which consumes a large portion of its dietary energy intake from wheat, fortifying flour would make sense. Another recent study reported that fortifying flour, oil, and milk with vitamins A, D, and E at levels suggested by recent international guidelines [159] would be cost-effective, with optimal intakes of vitamin D_3_ considered to be about 600 IU/day for males and 400 IU/day for females. A further study from those authors reported that 55% of rural and urban Mongolians favored the mandatory fortification of foods, 14% disapproved of it, and 31% were uncertain [160]. However, when the same people were informed that the primary purpose of adding vitamin D to milk was to prevent childhood rickets, 75% favored mandatory fortification. However, as estimated for the UK [158], while the postulated intake levels from fortification could overcome deficiency, they could not lead to sufficiency in Mongolians without concomitant supplementation of those at highest risk of deficiency.

### 4.3. Disclosure

This paper is an outcome of a visit to Ulaanbaatar, Mongolia, by WBG 15–21 April 2019, funded by Bayangol Med Co. Ltd., Mongolia, and Bio-Tech Pharmacal, Inc. (Fayetteville, AR, USA). WBG also receives funding for vitamin D research from Bio-Tech Pharmacal, Inc. BJB has no conflicts of interest to declare.

## Figures and Tables

**Table 1:** Age-standardized mortality rates for selected health outcomes in males and females in Mongolia in 2016 from the World Health Organization [13].

Outcome	Rate *	
	Males	Females
All causes	1244	712
Ischemic heart disease	322	191
Malignant neoplasms	275	162
Hemorrhagic stroke	183	122
Liver cancer	121	70
Tuberculosis	75	12
Cirrhosis of the liver	68	48
Stomach cancer	42	19
Trachea, bronchus, lung cancer	42	9
Ischemic stroke	23	14
Alzheimer’s disease	20	19
Kidney diseases	20	12
COPD	18	9
Lower respiratory tract infections	17	9
Hypertensive heart disease	11	7.5
Preterm birth complications	6.4	4.6
Diabetes mellitus	6.0	3.5
Measles	2.4	1.8

*, deaths/100,000/year; COPD, chronic obstructive pulmonary disease.

**Table 2:** Serum 25-hydroxyvitamin D (25(OH)D) concentrations as a function of occupation and season [16].

Occupation	25(OH)D (ng/mL) (mean (SD)	
	Summer	Winter
Outdoor	24 (7)	8 (3)
Indoor	21 (9)	8 (3)

**Table 3:** Selected age-standardized cancer incidence rates/100,000 population for 2008–2012 [65].

Cancer Type	Males	Females
	270	195
Liver (primary)	115	75
Cervix		25
Stomach	49	22
Esophagus	22	16
Breast		10
Lung	37	7
Colorectal	7	6
Ovary		6
Pancreas	5	4
Kidney	4	4

**Table 4:** Estimated reductions in age-standardized mortality rates with adequate supplementation for selected health outcomes in Mongolia in 2016 from the World Health Organization [13] according to findings from observational studies and clinical trials.

Outcome	Reduction by Vitamin D			Rate *	
	%	Males *	Females *	Males	Females
All causes	20	250 ± 125	140 ± 70	1244	712
Ischemic heart disease	18	60	35	322	191
Malignant neoplasms	15	40	25	275	162
Hemorrhagic stroke	0			183	122
Tuberculosis	0			75	12
Cirrhosis of the liver	20	15	10	68	48
Ischemic stroke	15	3	2	23	14
Alzheimer’s disease	0			20	19
Kidney diseases	0			20	12
COPD	0			18	9
Lower respiratory tract infections	20	3	2	17	9
Hypertensive heart disease	0			11	7.5
Preterm birth complications	30	2	1	6.4	4.6
Diabetes mellitus	25	2	1	6.0	3.5
Total of individual rates		125 ± 60	76 ± 40		

*, deaths/100,000/year; COPD, chronic obstructive pulmonary disease.

**Table 5:** 25(OH)D concentrations associated with optimal outcomes for various disorders.

Outcome	25(OH)D, ng/mL	Study Type	Ref.
All-cause mortality rate	36	Meta-analysis, observational	[9]
Ischemic heart disease	20	Meta-analysis, observational	[127]
Malignant neoplasms incidence	40	Open-label study	[79]
Hemorrhagic stroke	No data available		
Cirrhosis of the liver	No data available		
Ischemic stroke	15	Meta-analysis, observational	[24]
Alzheimer’s disease	20	Observational study	[128]
Kidney diseases	No data available		
COPD	20		[129]
Lower respiratory tract infections	10–20		[7]
Hypertensive heart disease	40	Open-label study	[45]
Preterm birth complications	40	Open-label study	[104]
Diabetes mellitus	40	RCT	[35]
Dental caries	20	Observational study	[113]
Falls, fractures	40	Review of RCTs	[130]

*, deaths/100,000/yr; COPD, chronic obstructive pulmonary disease; RCT: randomized controlled trial.

**Table 6:** Dietary supply of various food groups in Mongolia in 2013 from the Food and Agriculture Organization of the United Nations [14].

Item	Energy, kcal/capita/day
Wheat	961
Rice, other grains	27
Potatoes, other roots	99
Sugar and sweeteners	145
Nuts	12
Soybeans	55
Sunflowers (seeds)	53
Vegetable oils	49
Vegetables	39
Fruit	23
Cocoa beans	31
Beer, wine	45
Mutton	295
Other meat	290
Animal fat	81
Eggs	9
Milk	244
Butter	8
Fish	3
Miscellaneous	40
Total	2509

## References

[1] Webb A.R., Kline L., Holick M.F. (1988). Influence of Season and Latitude on the Cutaneous Synthesis of Vitamin D3: Exposure to Winter Sunlight in Boston and Edmonton Will Not Promote Vitamin D3 Synthesis in Human Skin. J. Clin. Endocrinol. Metab..

[2] Engelsen O. (2010). The Relationship between Ultraviolet Radiation Exposure and Vitamin D Status. Nutrients.

[3] World Bank Gdp Per Capita, Ppp (Current International $). https://data.worldbank.org/indicator/NY.GDP.PCAP.PP.CD?locations=UA-AM-GE-MN-AL&name_desc=false.

[4] Dugee O., Palam E., Dorjsuren B., Mahal A. (2018). Who Is Bearing the Financial Burden of Non-Communicable Diseases in Mongolia?. J. Glob. Health.

[5] Dugee O., Sugar B., Dorjsuren B., Mahal A. (2019). Economic Impacts of Chronic Conditions in a Country with High Levels of Population Health Coverage: Lessons from Mongolia. Trop. Med. Int. Health.

[6] Moukayed M., Grant W.B. (2013). Molecular Link between Vitamin D and Cancer Prevention. Nutrients.

[7] Martineau A.R., Jolliffe D.A., Hooper R.L., Greenberg L., Aloia J.F., Bergman P., Dubnov-Raz G., Esposito S., Ganmaa D., Ginde A.A. (2017). Vitamin D Supplementation to Prevent Acute Respiratory Tract Infections: Systematic Review and Meta-Analysis of Individual Participant Data. BMJ.

[8] Wagner C.L., Hollis B.W. (2018). The Implications of Vitamin D Status During Pregnancy on Mother and Her Developing Child. Front. Endocrinol..

[9] Garland C.F., Kim J.J., Mohr S.B., Gorham E.D., Grant W.B., Giovannucci E.L., Baggerly L., Hofflich H., Ramsdell J.W., Zeng K. (2014). Meta-Analysis of All-Cause Mortality According to Serum 25-Hydroxyvitamin D. Am. J. Public Health.

[10] Holick M.F. (2007). Vitamin D Deficiency. N. Engl. J. Med..

[11] Pludowski P., Holick M.F., Pilz S., Wagner C.L., Hollis B.W., Grant W.B., Shoenfeld Y., Lerchbaum E., Llewellyn D.J., Kienreich K. (2013). Vitamin D Effects on Musculoskeletal Health, Immunity, Autoimmunity, Cardiovascular Disease, Cancer, Fertility, Pregnancy, Dementia and Mortality—A Review of Recent Evidence. Autoimmun. Rev..

[12] Wimalawansa S.J. (2018). Non-Musculoskeletal Benefits of Vitamin D. J. Steroid Biochem. Mol. Biol..

[13] World Health Organization Global Health Estimates 2016: Deaths by Cause, Age, Sex, by Country and by Region, 2000–2016. https://www.who.int/healthinfo/global_burden_disease/GHE2016_Death-Rates-country.xls?ua=1.

[14] FAO Food Balance Sheets. Food and Agriculture Organization of the United Nations..

[15] FAO (2011). Global Food Losses and Food Waste—Extent, Causes and Prevention.

[16] Bromage S., Rich-Edwards J.W., Tselmen D., Baylin A., Houghton L.A., Baasanjav N., Ganmaa D. (2016). Seasonal Epidemiology of Serum 25-Hydroxyvitamin D Concentrations among Healthy Adults Living in Rural and Urban Areas in Mongolia. Nutrients.

[17] Dunlop E., Cunningham J., Sherriff J.L., Lucas R.M., Greenfield H., Arcot J., Strobel N., Black L.J. (2017). Vitamin D(3) and 25-Hydroxyvitamin D(3) Content of Retail White Fish and Eggs in Australia. Nutrients.

[18] Fraser D.R. (2004). Vitamin D-Deficiency in Asia. J. Steroid Biochem. Mol. Biol..

[19] Uush T. (2013). Prevalence of Classic Signs and Symptoms of Rickets and Vitamin D Deficiency in Mongolian Children and Women. J. Steroid Biochem. Mol. Biol..

[20] Brondum-Jacobsen P., Benn M., Jensen G.B., Nordestgaard B.G. (2012). 25-Hydroxyvitamin D Levels and Risk of Ischemic Heart Disease, Myocardial Infarction, and Early Death: Population-Based Study and Meta-Analyses of 18 and 17 Studies. Arterioscler. Thromb. Vasc. Biol..

[21] Lee K.J., Lee Y.J. (2016). Effects of Vitamin D on Blood Pressure in Patients with Type 2 Diabetes Mellitus. Int. J. Clin. Pharmacol. Ther..

[22] Barbarawi M., Kheiri B., Zayed Y., Barbarawi O., Dhillon H., Swaid B., Yelangi A., Sundus S., Bachuwa G., Alkotob M.L. (2019). Vitamin D Supplementation and Cardiovascular Disease Risks in More Than 83000 Individuals in 21 Randomized Clinical Trials: A Meta-Analysis. JAMA Cardiol..

[23] Manson J.E., Cook N.R., Lee I.M., Christen W., Bassuk S.S., Mora S., Gibson H., Gordon D., Copeland T., D’Agostino D. (2019). Vitamin D Supplements and Prevention of Cancer and Cardiovascular Disease. N. Engl. J. Med..

[24] Zhou R., Wang M., Huang H., Li W., Hu Y., Wu T. (2018). Lower Vitamin D Status Is Associated with an Increased Risk of Ischemic Stroke: A Systematic Review and Meta-Analysis. Nutrients.

[25] Alfieri D.F., Lehmann M.F., Oliveira S.R., Flauzino T., Delongui F., de Araujo M.C., Dichi I., Delfino V.D., Mezzaroba L., Simao A.N. (2017). Vitamin D Deficiency Is Associated with Acute Ischemic Stroke, C-Reactive Protein, and Short-Term Outcome. Metab. Brain Dis..

[26] Hypponen E., Boucher B.J. (2018). Adiposity, Vitamin D Requirements, and Clinical Implications for Obesity-Related Metabolic Abnormalities. Nutr. Rev..

[27] Mousavi S.E., Amini H., Heydarpour P., Chermahini F.A., Godderis L. (2019). Air Pollution, Environmental Chemicals, and Smoking May Trigger Vitamin D Deficiency: Evidence and Potential Mechanisms. Environ. Int..

[28] Kojima G., Bell C., Abbott R.D., Launer L., Chen R., Motonaga H., Ross G.W., Curb J.D., Masaki K. (2012). Low Dietary Vitamin D Predicts 34-Year Incident Stroke: The Honolulu Heart Program. Stroke.

[29] Venketasubramanian N., Yoon B.W., Pandian J., Navarro J.C. (2017). Stroke Epidemiology in South, East, and South-East Asia: A Review. J. Stroke.

[30] The Tobacco Atlas American Cancer Society. https://tobaccoatlas.org/country/mongolia/.

[31] Forouhi N.G., Luan J., Cooper A., Boucher B.J., Wareham N.J. (2008). Baseline Serum 25-Hydroxy Vitamin D Is Predictive of Future Glycemic Status and Insulin Resistance: The Medical Research Council Ely Prospective Study 1990–2000. Diabetes.

[32] Lucato P., Solmi M., Maggi S., Bertocco A., Bano G., Trevisan C., Manzato E., Sergi G., Schofield P., Kouidrat Y. (2017). Low Vitamin D Levels Increase the Risk of Type 2 Diabetes in Older Adults: A Systematic Review and Meta-Analysis. Maturitas.

[33] Rafiq S., Jeppesen P.B. (2018). Is Hypovitaminosis D Related to Incidence of Type 2 Diabetes and High Fasting Glucose Level in Healthy Subjects: A Systematic Review and Meta-Analysis of Observational Studies. Nutrients.

[34] Niroomand M., Fotouhi A., Irannejad N., Hosseinpanah F. (2019). Does High-Dose Vitamin D Supplementation Impact Insulin Resistance and Risk of Development of Diabetes in Patients with Pre-Diabetes? A Double-Blind Randomized Clinical Trial. Diabetes Res. Clin. Pract..

[35] Pittas A.G., Dawson-Hughes B., Sheehan P., Ware J.H., Knowler W.C., Aroda V.R., Brodsky I., Ceglia L., Chadha C., Chatterjee R. (2019). Vitamin D Supplementation and Prevention of Type 2 Diabetes. N. Engl. J. Med..

[36] Lemieux P., Weisnagel J.S., Caron A.Z., Julien A.S., Morisset A.S., Carreau A.M., Poirier J., Tchernof A., Robitaille J., Bergeron J. (2019). Effects of 6-Month Vitamin D Supplementation on Insulin Sensitivity and Secretion: A Randomized, Placebo-Controlled Trial. Eur. J. Endocrinol..

[37] Bo Y., Guo C., Lin C., Chang L.Y., Chan T.C., Huang B., Lee K.P., Tam T., Lau A.K.H., Lao X.Q. (2019). Dynamic Changes in Long-Term Exposure to Ambient Particulate Matter and Incidence of Hypertension in Adults. Hypertension.

[38] Fukuoka T., Nakazato Y., Kawasaki H., Ikeda K., Furuya T., Miyake A., Mitsufuji T., Ito Y., Takahashi K., Araki N. (2018). The Clinical Features of Ischemic Stroke Patients for Whom Smoking Was Considered the Sole Risk Factor for Ischemic Stroke. Intern. Med..

[39] Fujihara Y., Nawata H., Honda M., Kunitake T., Aida E., Nagai T., Kuramochi H., Ueno J., Yoshimoto S., Muta K. (2017). Comparative Study of the Correlation between Atherosclerosis and Osteoporosis in Women in Japan and Mongolia. J. Gen. Fam. Med..

[40] Myanganbayar M., Baatarsuren U., Chen G., Bosurgi R., So G., Campbell N.R.C., Erdenebileg N., Ganbaatar K., Magsarjav P., Batsukh M. (2018). Hypertension, Knowledge, Attitudes, and Practices of Primary Care Physicians in Ulaanbaatar, Mongolia. J. Clin. Hypertens..

[41] Myanganbayar M., Baatarsuren U., Chen G., Campbell N.R.C., Bosurgi R., So G., Unurjargal T., Dashtseren M., Tserengombo N., Batsukh B. (2019). Hypertension Knowledge, Attitudes, and Practices of Nurses and Physicians in Primary Care in Ulaanbaatar Mongolia. J. Clin. Hypertens..

[42] Lin L., Zhang L., Li C., Gai Z., Li Y. (2019). Vitamin D and Vitamin D Receptor: New Insights in the Treatment of Hypertension. Curr. Protein. Pept. Sci..

[43] Tabas I. (2017). 2016 Russell Ross Memorial Lecture in Vascular Biology: Molecular-Cellular Mechanisms in the Progression of Atherosclerosis. Arterioscler. Thromb. Vasc. Biol..

[44] He S., Hao X. (2019). The Effect of Vitamin D3 on Blood Pressure in People with Vitamin D Deficiency: A System Review and Meta-Analysis. Medicine.

[45] Mirhosseini N., Vatanparast H., Kimball S.M. (2017). The Association between Serum 25(Oh)D Status and Blood Pressure in Participants of a Community-Based Program Taking Vitamin D Supplements. Nutrients.

[46] Hamrahian S.M., Falkner B. (2017). Hypertension in Chronic Kidney Disease. Adv. Exp. Med. Biol..

[47] Jean G., Souberbielle J.C., Chazot C. (2017). Vitamin D in Chronic Kidney Disease and Dialysis Patients. Nutrients.

[48] Adams J.S., Hewison M. (2012). Extrarenal Expression of the 25-Hydroxyvitamin D-1-Hydroxylase. Arch. Biochem. Biophys..

[49] Liu W.C., Wu C.C., Hung Y.M., Liao M.T., Shyu J.F., Lin Y.F., Lu K.C., Yeh K.C. (2016). Pleiotropic Effects of Vitamin D in Chronic Kidney Disease. Clin. Chim. Acta.

[50] Lu R.J., Zhu S.M., Tang F.L., Zhu X.S., Fan Z.D., Wang G.L., Jiang Y.F., Zhang Y. (2017). Effects of Vitamin D or Its Analogues on the Mortality of Patients with Chronic Kidney Disease: An Updated Systematic Review and Meta-Analysis. Eur. J. Clin. Nutr..

[51] Molina P., Gorriz J.L., Molina M.D., Beltran S., Vizcaino B., Escudero V., Kanter J., Avila A.I., Bover J., Fernandez E. (2016). What Is the Optimal Level of Vitamin D in Non-Dialysis Chronic Kidney Disease Population?. World J. Nephrol..

[52] Zhu M., Wang T., Wang C., Ji Y. (2016). The Association between Vitamin D and Copd Risk, Severity, and Exacerbation: An Updated Systematic Review and Meta-Analysis. Int. J. Chron. Obstr. Pulm. Dis..

[53] Jolliffe D.A., Greenberg L., Hooper R.L., Mathyssen C., Rafiq R., de Jongh R.T., Camargo C.A., Griffiths C.J., Janssens W., Martineau A.R. (2019). Vitamin D to Prevent Exacerbations of Copd: Systematic Review and Meta-Analysis of Individual Participant Data from Randomised Controlled Trials. Thorax.

[54] Kokturk N., Baha A., Oh Y.M., Ju J.Y., Jones P.W. (2018). Vitamin D Deficiency: What Does It Mean for Chronic Obstructive Pulmonary Disease (Copd)? A Compherensive Review for Pulmonologists. Clin. Respir. J..

[55] Tao S., Zhang H., Xue L., Jiang X., Wang H., Li B., Tian H., Zhang Z. (2019). Vitamin D Protects against Particles-Caused Lung Injury through Induction of Autophagy in an Nrf2-Dependent Manner. Environ. Toxicol..

[56] Cannell J.J., Vieth R., Umhau J.C., Holick M.F., Grant W.B., Madronich S., Garland C.F., Giovannucci E. (2006). Epidemic Influenza and Vitamin D. Epidemiol. Infect..

[57] Aloia J.F., Li-Ng M. (2007). Re: Epidemic Influenza and Vitamin D. Epidemiol. Infect..

[58] Urashima M., Segawa T., Okazaki M., Kurihara M., Wada Y., Ida H. (2010). Randomized Trial of Vitamin D Supplementation to Prevent Seasonal Influenza a in Schoolchildren. Am. J. Clin. Nutr..

[59] Camargo C.A., Ganmaa D., Frazier A.L., Kirchberg F.F., Stuart J.J., Kleinman K., Sumberzul N., Rich-Edwards J.W. (2012). Randomized Trial of Vitamin D Supplementation and Risk of Acute Respiratory Infection in Mongolia. Pediatrics.

[60] Martineau A.R., Jolliffe D.A., Greenberg L., Aloia J.F., Bergman P., Dubnov-Raz G., Esposito S., Ganmaa D., Ginde A.A., Goodall E.C. (2019). Vitamin D Supplementation to Prevent Acute Respiratory Infections: Individual Participant Data Meta-Analysis. Health Technol. Assess..

[61] Grant W.B., Giovannucci E. (2009). The Possible Roles of Solar Ultraviolet-B Radiation and Vitamin D in Reducing Case-Fatality Rates from the 1918–1919 Influenza Pandemic in the United States. Dermatoendocrinol.

[62] Mehta S., Mugusi F.M., Bosch R.J., Aboud S., Urassa W., Villamor E., Fawzi W.W. (2013). Vitamin D Status and Tb Treatment Outcomes in Adult Patients in Tanzania: A Cohort Study. BMJ Open.

[63] Ganmaa D., Munkhzul B., Fawzi W., Spiegelman D., Willett W.C., Bayasgalan P., Baasansuren E., Buyankhishig B., Oyun-Erdene S., Jolliffe D.A. (2017). High-Dose Vitamin D3 During Tuberculosis Treatment in Mongolia. A Randomized Controlled Trial. Am. J. Respir. Crit. Care Med..

[64] Ganmaa D., Khudyakov P., Buyanjargal U., Baigal D., Baatar M., Enkhamgalan N., Erdenebaatar S., Ochirbat B., Burneebaatar B., Purevdorj E. (2019). Risk Factors for Active Tuberculosis in 938 Quantiferon-Positive Schoolchildren in Mongolia: A Community-Based Cross-Sectional Study. BMC Infect. Dis..

[65] Chimed T., Sandagdorj T., Znaor A., Laversanne M., Tseveen B., Genden P., Bray F. (2017). Cancer Incidence and Cancer Control in Mongolia: Results from the National Cancer Registry 2008-12. Int. J. Cancer.

[66] Moukayed M., Grant W.B. (2017). The Roles of Uvb and Vitamin D in Reducing Risk of Cancer Incidence and Mortality: A Review of the Epidemiology, Clinical Trials, and Mechanisms. Rev. Endocr. Metab. Disord..

[67] Grant W.B., Boucher B.J. (2017). Randomized Controlled Trials of Vitamin D and Cancer Incidence: A Modeling Study. PLoS ONE.

[68] McDonnell S.L., Baggerly C.A., French C.B., Baggerly L.L., Garland C.F., Gorham E.D., Hollis B.W., Trump D.L., Lappe J.M. (2018). Breast Cancer Risk Markedly Lower with Serum 25-Hydroxyvitamin D Concentrations >/=60 Vs <20 Ng/Ml (150 Vs 50 Nmol/L): Pooled Analysis of Two Randomized Trials and a Prospective Cohort. PLoS ONE.

[69] McCullough M.L., Zoltick E.S., Weinstein S.J., Fedirko V., Wang M., Cook N.R., Eliassen A.H., Zeleniuch-Jacquotte A., Agnoli C., Albanes D. (2019). Circulating Vitamin D and Colorectal Cancer Risk: An International Pooling Project of 17 Cohorts. J. Natl. Cancer Inst..

[70] Zhang Y., Fang F., Tang J., Jia L., Feng Y., Xu P., Faramand A. (2019). Association between Vitamin D Supplementation and Mortality: Systematic Review and Meta-Analysis. BMJ.

[71] O’Sullivan F., Laird E., Kelly D., van Geffen J., van Weele M., McNulty H., Hoey L., Healy M., McCarroll K., Cunningham C. (2017). Ambient Uvb Dose and Sun Enjoyment Are Important Predictors of Vitamin D Status in an Older Population. J. Nutr..

[72] Tran B., Lucas R., Kimlin M., Whiteman D., Neale R. (2012). Association between Ambient Ultraviolet Radiation and Risk of Esophageal Cancer. Am. J. Gastroenterol..

[73] Du C., Yang S., Zhao X., Dong H. (2017). Pathogenic Roles of Alterations in Vitamin D and Vitamin D Receptor in Gastric Tumorigenesis. Oncotarget.

[74] El Shahawy M.S., Hemida M.H., El Metwaly I., Shady Z.M. (2018). The Effect of Vitamin D Deficiency on Eradication Rates of Helicobacter Pylori Infection. JGH Open.

[75] Han C., Ni Z., Yuan T., Zhang J., Wang C., Wang X., Ning H.B., Liu J., Sun N., Liu C.F. (2019). Influence of Serum Vitamin D Level on Helicobacter Pylori Eradication: A Multi-Center, Observational, Prospective and Cohort Study. J. Dig. Dis..

[76] Fedirko V., Duarte-Salles T., Bamia C., Trichopoulou A., Aleksandrova K., Trichopoulos D., Trepo E., Tjonneland A., Olsen A., Overvad K. (2014). Prediagnostic Circulating Vitamin D Levels and Risk of Hepatocellular Carcinoma in European Populations: A Nested Case-Control Study. Hepatology.

[77] Manios Y., Moschonis G., Lambrinou C.P., Mavrogianni C., Tsirigoti L., Hoeller U., Roos F.F., Bendik I., Eggersdorfer M., Celis-Morales C. (2018). Associations of Vitamin D Status with Dietary Intakes and Physical Activity Levels among Adults from Seven European Countries: The Food4me Study. Eur. J. Nutr..

[78] Budhathoki S., Hidaka A., Yamaji T., Sawada N., Tanaka-Mizuno S., Kuchiba A., Charvat H., Goto A., Kojima S., Sudo N. (2018). Plasma 25-Hydroxyvitamin D Concentration and Subsequent Risk of Total and Site Specific Cancers in Japanese Population: Large Case-Cohort Study within Japan Public Health Center-Based Prospective Study Cohort. BMJ.

[79] McDonnell S.L., Baggerly C., French C.B., Baggerly L.L., Garland C.F., Gorham E.D., Lappe J.M., Heaney R.P. (2016). Serum 25-Hydroxyvitamin D Concentrations >/=40 Ng/Ml Are Associated with >65% Lower Cancer Risk: Pooled Analysis of Randomized Trial and Prospective Cohort Study. PLoS ONE.

[80] Grant W.B., Boucher B.J. (2019). Why Secondary Analyses in Vitamin D Clinical Trials Are Important and How to Improve Vitamin D Clinical Trial Outcome Analyses—A Comment on “Extra-Skeletal Effects of Vitamin D, Nutrients 2019, 11, 1460”. Nutrients.

[81] Jazag A., Puntsagdulam N., Chinburen J. (2012). Status Quo of Chronic Liver Diseases, Including Hepatocellular Carcinoma, in Mongolia. Korean J. Int. Med..

[82] Kim H.B., Myung S.K., Lee Y.J., Park B.J., Group Korean Meta-Analysis Study (2018). Efficacy of Vitamin D Supplementation in Combination with Conventional Antiviral Therapy in Patients with Chronic Hepatitis C Infection: A Meta-Analysis of Randomised Controlled Trials. J. Hum. Nutr. Diet..

[83] Jin C.N., Chen J.D., Sheng J.F. (2018). Vitamin D Deficiency in Hepatitis C Virus Infection: What Is Old? What Is New?. Eur. J. Gastroenterol. Hepatol..

[84] Hernandez-Alvarez N., Acevedo J.M.P., Quintero E., Vazquez I.F., Garcia-Eliz M., Negro J.D., Garcia J.C., Hernandez-Guerra M. (2017). Effect of Season and Sunlight on Viral Kinetics During Hepatitis C Virus Therapy. BMJ Open Gastroenterol..

[85] Yang F., Ren H., Gao Y., Zhu Y., Huang W. (2019). The Value of Severe Vitamin D Deficiency in Predicting the Mortality Risk of Patients with Liver Cirrhosis: A Meta-Analysis. Clin. Res. Hepatol. Gastroenterol..

[86] Mosconi L., Murray J., Davies M., Williams S., Pirraglia E., Spector N., Tsui W.H., Li Y., Butler T., Osorio R.S. (2014). Nutrient Intake and Brain Biomarkers of Alzheimer’s Disease in at-Risk Cognitively Normal Individuals: A Cross-Sectional Neuroimaging Pilot Study. BMJ Open.

[87] Grant W.B. (2016). Using Multicountry Ecological and Observational Studies to Determine Dietary Risk Factors for Alzheimer’s Disease. J. Am. Coll. Nutr..

[88] Huriletemuer H., Wen S., Zhang C., Zhao S., Niu G., Wang B., Ma X., Wang D. (2011). An Epidemiological Study of Alzheimer’s Disease in Elderly Mongolian and Han Populations Living in Rural Areas of Inner Mongolia. Aging Clin. Exp. Res..

[89] Feart C., Helmer C., Merle B., Herrmann F.R., Annweiler C., Dartigues J.F., Delcourt C., Samieri C. (2017). Associations of Lower Vitamin D Concentrations with Cognitive Decline and Long-Term Risk of Dementia and Alzheimer’s Disease in Older Adults. Alzheimers Dement..

[90] Liu J., Greenfield H., Strobel N., Fraser D.R. (2013). The Influence of Latitude on the Concentration of Vitamin D3 and 25-Hydroxy-Vitamin D3 in Australian Red Meat. Food Chem..

[91] Calderon-Garciduenas L., Franco-Lira M., D’Angiulli A., Rodriguez-Diaz J., Blaurock-Busch E., Busch Y., Chao C.K., Thompson C., Mukherjee P.S., Torres-Jardon R. (2015). Mexico City Normal Weight Children Exposed to High Concentrations of Ambient Pm2.5 Show High Blood Leptin and Endothelin-1, Vitamin D Deficiency, and Food Reward Hormone Dysregulation Versus Low Pollution Controls. Relevance for Obesity and Alzheimer Disease. Environ. Res..

[92] Manousaki D., Mokry L.E., Ross S., Goltzman D., Richards J.B. (2016). Mendelian Randomization Studies Do Not Support a Role for Vitamin D in Coronary Artery Disease. Circ. Cardiovasc. Genet..

[93] Dursun E., Gezen-Ak D. (2019). Vitamin D Basis of Alzheimer’s Disease: From Genetics to Biomarkers. Hormones.

[94] Enkhmaa D., Warburton N., Javzandulam B., Uyanga J., Khishigsuren Y., Lodoysamba S., Enkhtur S., Warburton D. (2014). Seasonal Ambient Air Pollution Correlates Strongly with Spontaneous Abortion in Mongolia. BMC Pregnancy Childbirth.

[95] Bove H., Bongaerts E., Slenders E., Bijnens E.M., Saenen N.D., Gyselaers W., van Eyken P., Plusquin M., Roeffaers M.B.J., Ameloot M. (2019). Ambient Black Carbon Particles Reach the Fetal Side of Human Placenta. Nat. Commun..

[96] Momentti A.C., Estadella D., Pisani L.P. (2018). Role of Vitamin D in Pregnancy and Toll-Like Receptor Pathway. Steroids.

[97] Hou W., Yan X.T., Bai C.M., Zhang X.W., Hui L.Y., Yu X.W. (2016). Decreased Serum Vitamin D Levels in Early Spontaneous Pregnancy Loss. Eur. J. Clin. Nutr..

[98] Karras S.N., Polyzos S.A., Newton D.A., Wagner C.L., Hollis B.W., Ouweland J.V.D., Dursun E., Gezen-Ak D., Kotsa K., Annweiler C. (2018). Adiponectin and Vitamin D-Binding Protein Are Independently Associated at Birth in Both Mothers and Neonates. Endocrine.

[99] Luxwolda M.F., Kuipers R.S., Kema I.P., Dijck-Brouwer D.A., Muskiet F.A. (2012). Traditionally Living Populations in East Africa Have a Mean Serum 25-Hydroxyvitamin D Concentration of 115 Nmol/L. Br. J. Nutr..

[100] Shen Y., Pu L., Si S., Xin X., Mo M., Shao B., Wu J., Huang M., Wang S., Muyiduli X. (2019). Vitamin D Nutrient Status During Pregnancy and Its Influencing Factors. Clin. Nutr..

[101] Marchand N.E., Davaasambuu G., McElrath T.F., Davaasambuu E., Baatar T., Troisi R. (2016). Prevalence of Pregnancy Hypertensive Disorders in Mongolia. Pregnancy Hypertens.

[102] Bilano V.L., Ota E., Ganchimeg T., Mori R., Souza J.P. (2014). Risk Factors of Pre-Eclampsia/Eclampsia and Its Adverse Outcomes in Low- and Middle-Income Countries: A Who Secondary Analysis. PLoS ONE.

[103] Morisaki N., Ganchimeg T., Vogel J.P., Zeitlin J., Cecatti J.G., Souza J.P., Castro C.P., Torloni M.R., Ota E., Mori R. (2017). Research Network the. Impact of Stillbirths on International Comparisons of Preterm Birth Rates: A Secondary Analysis of the Who Multi-Country Survey of Maternal and Newborn Health. BJOG.

[104] McDonnell S.L., Baggerly K.A., Baggerly C.A., Aliano J.L., French C.B., Baggerly L.L., Ebeling M.D., Rittenberg C.S., Goodier C.G., Nino J.F.M. (2017). Maternal 25(Oh)D Concentrations >/=40 Ng/Ml Associated with 60% Lower Preterm Birth Risk among General Obstetrical Patients at an Urban Medical Center. PLoS ONE.

[105] Xia J., Song Y., Rawal S., Wu J., Hinkle S.N., Tsai M.Y., Zhang C. (2019). Vitamin D Status During Pregnancy and the Risk of Gestational Diabetes Mellitus: A Longitudinal Study in a Multiracial Cohort. Diabetes Obes. Metab..

[106] Jamilian M., Mirhosseini N., Eslahi M., Bahmani F., Shokrpour M., Chamani M., Asemi Z. (2019). The Effects of Magnesium-Zinc-Calcium-Vitamin D Co-Supplementation on Biomarkers of Inflammation, Oxidative Stress and Pregnancy Outcomes in Gestational Diabetes. BMC Pregnancy Childbirth.

[107] Enkhmaa D., Tanz L., Ganmaa D., Enkhtur S., Oyun-Erdene B., Stuart J., Chen G., Carr A., Seely E.W., Fitzmaurice G. (2019). Randomized Trial of Three Doses of Vitamin D to Reduce Deficiency in Pregnant Mongolian Women. EBioMedicine.

[108] Mellanby M., Pattison C.L. (1928). The Action of Vitamin D in Preventing the Spread and Promoting the Arrest of Caries in Children. Br. Med. J..

[109] Grant W.B. (2011). A Review of the Role of Solar Ultraviolet-B Irradiance and Vitamin D in Reducing Risk of Dental Caries. Dermatoendocrinol.

[110] Van Nieuw Amerongen A., Bolscher J.G., Veerman E.C. (2004). Salivary Proteins: Protective and Diagnostic Value in Cariology?. Caries. Res..

[111] Liu P.T., Stenger S., Li H., Wenzel L., Tan B.H., Krutzik S.R., Ochoa M.T., Schauber J., Wu K., Meinken C. (2006). Toll-Like Receptor Triggering of a Vitamin D-Mediated Human Antimicrobial Response. Science.

[112] Hujoel P.P. (2013). Vitamin D and Dental Caries in Controlled Clinical Trials: Systematic Review and Meta-Analysis. Nutr. Rev..

[113] Gyll J., Ridell K., Ohlund I., Akeson P.K., Johansson I., Holgerson P.L. (2018). Vitamin D Status and Dental Caries in Healthy Swedish Children. Nutr. J..

[114] Grant W.B., Boucher B.J. (2010). Are Hill’s Criteria for Causality Satisfied for Vitamin D and Periodontal Disease?. Dermatoendocrinol.

[115] Khammissa R.A.G., Ballyram R., Jadwat Y., Fourie J., Lemmer J., Feller L. (2018). Vitamin D Deficiency as It Relates to Oral Immunity and Chronic Periodontitis. Int. J. Dent..

[116] Jagelaviciene E., Vaitkeviciene I., Silingaite D., Sinkunaite E., Daugelaite G. (2018). The Relationship between Vitamin D and Periodontal Pathology. Medicina.

[117] Adegboye A.R., Boucher B.J., Kongstad J., Fiehn N.E., Christensen L.B., Heitmann B.L. (2016). Calcium, Vitamin D, Casein and Whey Protein Intakes and Periodontitis among Danish Adults. Public Health Nutr..

[118] Aspelund T., Grubler M.R., Smith A.V., Gudmundsson E.F., Keppel M., Cotch M.F., Harris T.B., Jorde R., Grimnes G., Joakimsen R. (2019). Effect of Genetically Low 25-Hydroxyvitamin D on Mortality Risk: Mendelian Randomization Analysis in 3 Large European Cohorts. Nutrients.

[119] Mao C., Li F.R., Yin Z.X., Lv Y.B., Luo J.S., Yuan J.Q., Mhungu F., Wang J.N., Shi W.Y., Zhou J.H. (2019). Plasma 25-Hydroxyvitamin D Concentrations Are Inversely Associated with All-Cause Mortality among a Prospective Cohort of Chinese Adults Aged >/=80 Years. J. Nutr..

[120] Lindqvist P.G., Epstein E., Nielsen K., Landin-Olsson M., Ingvar C., Olsson H. (2016). Avoidance of Sun Exposure as a Risk Factor for Major Causes of Death: A Competing Risk Analysis of the Melanoma in Southern Sweden Cohort. J. Int. Med..

[121] Grant W.B. (2011). An Estimate of the Global Reduction in Mortality Rates through Doubling Vitamin D Levels. Eur. J. Clin. Nutr..

[122] Grant W.B., Whiting S.J., Schwalfenberg G.K., Genuis S.J., Kimball S.M. (2016). Estimated Economic Benefit of Increasing 25-Hydroxyvitamin D Concentrations of Canadians to or above 100 Nmol/L. Dermatoendocrinol.

[123] Autier P., Boniol M., Pizot C., Mullie P. (2014). Vitamin D Status and Ill Health: A Systematic Review. Lancet Diabetes Endocrinol..

[124] Theodoratou E., Tzoulaki I., Zgaga L., Ioannidis J.P. (2014). Vitamin D and Multiple Health Outcomes: Umbrella Review of Systematic Reviews and Meta-Analyses of Observational Studies and Randomised Trials. BMJ.

[125] Christakos S., Dhawan P., Verstuyf A., Verlinden L., Carmeliet G. (2016). Vitamin D: Metabolism, Molecular Mechanism of Action, and Pleiotropic Effects. Physiol. Rev..

[126] Haussler M.R., Whitfield G.K., Kaneko I., Haussler C.A., Hsieh D., Hsieh J.C., Jurutka P.W. (2013). Molecular Mechanisms of Vitamin D Action. Calcif. Tissue Int..

[127] Zhang R., Li B., Gao X., Tian R., Pan Y., Jiang Y., Gu H., Wang Y., Liu G. (2017). Serum 25-Hydroxyvitamin D and the Risk of Cardiovascular Disease: Dose-Response Meta-Analysis of Prospective Studies. Am. J. Clin. Nutr..

[128] Littlejohns T.J., Henley W.E., Lang I.A., Annweiler C., Beauchet O., Chaves P.H., Fried L., Kestenbaum B.R., Kuller L.H., Langa K.M. (2014). Vitamin D and the Risk of Dementia and Alzheimer Disease. Neurology.

[129] Faerk G., Colak Y., Afzal S., Nordestgaard B.G. (2018). Low Concentrations of 25-Hydroxyvitamin D and Long-Term Prognosis of Copd: A Prospective Cohort Study. Eur. J. Epidemiol..

[130] Bischoff-Ferrari H.A. (2012). Vitamin D and Fracture Prevention. Rheum. Dis. Clin. N. Am..

[131] Wolman R., Wyon M.A., Koutedakis Y., Nevill A.M., Eastell R., Allen N. (2013). Vitamin D Status in Professional Ballet Dancers: Winter Vs. Summer. J. Sci. Med. Sport.

[132] Godar D.E., Pope S.J., Grant W.B., Holick M.F. (2011). Solar Uv Doses of Adult Americans and Vitamin D(3) Production. Dermatoendocrinol.

[133] Hill L.D., Edwards R., Turner J.R., Argo Y.D., Olkhanud P.B., Odsuren M., Guttikunda S., Ochir C., Smith K.R. (2017). Health Assessment of Future Pm2.5 Exposures from Indoor, Outdoor, and Secondhand Tobacco Smoke Concentrations under Alternative Policy Pathways in Ulaanbaatar, Mongolia. PLoS ONE.

[134] Burnett A., Davey C.G., Wood S.J., Wilson-Ching M., Molloy C., Cheong J.L., Doyle L.W., Anderson P.J. (2014). Extremely Preterm Birth and Adolescent Mental Health in a Geographical Cohort Born in the 1990s. Psychol. Med..

[135] Alberg A.J., Shopland D.R., Cummings K.M. (2014). The 2014 Surgeon General’s Report: Commemorating the 50th Anniversary of the 1964 Report of the Advisory Committee to the Us Surgeon General and Updating the Evidence on the Health Consequences of Cigarette Smoking. Am. J. Epidemiol..

[136] Lee H., Kim K.N., Lim Y.H., Hong Y.C. (2015). Interaction of Vitamin D and Smoking on Inflammatory Markers in the Urban Elderly. J. Prev. Med. Public Health.

[137] Dodds L., Woolcott C.G., Weiler H., Spencer A., Forest J.C., Armson B.A., Giguere Y. (2016). Vitamin D Status and Gestational Diabetes: Effect of Smoking Status During Pregnancy. Paediatr. Perinat. Epidemiol..

[138] Heaney R.P. (2014). Guidelines for Optimizing Design and Analysis of Clinical Studies of Nutrient Effects. Nutr. Rev..

[139] Grant W.B., Boucher B.J., Bhattoa H.P., Lahore H. (2018). Why Vitamin D Clinical Trials Should Be Based on 25-Hydroxyvitamin D Concentrations. J. Steroid Biochem. Mol. Biol..

[140] Grant W.B. (2012). Effect of Follow-up Time on the Relation between Prediagnostic Serum 25-Hydroxyvitamin D and All-Cause Mortality Rate. Dermatoendocrinol.

[141] Grant W.B. (2015). 25-Hydroxyvitamin D and Breast Cancer, Colorectal Cancer, and Colorectal Adenomas: Case-Control Versus Nested Case-Control Studies. Anticancer Res..

[142] Crowe F.L., Steur M., Allen N.E., Appleby P.N., Travis R.C., Key T.J. (2011). Plasma Concentrations of 25-Hydroxyvitamin D in Meat Eaters, Fish Eaters, Vegetarians and Vegans: Results from the Epic-Oxford Study. Public Health Nutr..

[143] Liu J., Arcot J., Cunningham J., Greenfield H., Hsu J., Padula D., Strobel N., Fraser D.R. (2015). New Data for Vitamin D in Australian Foods of Animal Origin: Impact on Estimates of National Adult Vitamin D Intakes in 1995 and 2011-13. Asia Pac. J. Clin. Nutr..

[144] Heaney R.P., Davies K.M., Chen T.C., Holick M.F., Barger-Lux M.J. (2003). Human Serum 25-Hydroxycholecalciferol Response to Extended Oral Dosing with Cholecalciferol. Am. J. Clin. Nutr..

[145] Pludowski P., Holick M.F., Grant W.B., Konstantynowicz J., Mascarenhas M.R., Haq A., Povoroznyuk V., Balatska N., Barbosa A.P., Karonova T. (2018). Vitamin D Supplementation Guidelines. J. Steroid Biochem. Mol. Biol..

[146] De Pergola G., Martino T., Zupo R., Caccavo D., Pecorella C., Paradiso S., Silvestris F., Triggiani V. (2019). 25 Hydroxyvitamin D Levels Are Negatively and Independently Associated with Fat Mass in a Cohort of Healthy Overweight and Obese Subjects. Endocr. Metab. Immune Disord. Drug. Targets.

[147] Drincic A.T., Armas L.A., van Diest E.E., Heaney R.P. (2012). Volumetric Dilution, Rather Than Sequestration Best Explains the Low Vitamin D Status of Obesity. Obesity.

[148] Ross A.C., Manson J.E., Abrams S.A., Aloia J.F., Brannon P.M., Clinton S.K., Durazo-Arvizu R.A., Gallagher J.C., Gallo R.L., Jones G. (2011). The 2011 Report on Dietary Reference Intakes for Calcium and Vitamin D from the Institute of Medicine: What Clinicians Need to Know. J. Clin. Endocrinol. Metab..

[149] Holick M.F., Binkley N.C., Bischoff-Ferrari H.A., Gordon C.M., Hanley D.A., Heaney R.P., Murad M.H., Weaver C.M. (2011). Evaluation, Treatment, and Prevention of Vitamin D Deficiency: An Endocrine Society Clinical Practice Guideline. J. Clin. Endocrinol. Metab..

[150] Holick M.F. (2002). The Underappreciated D-Lightful Hormone That Is Important for Skeletal and Cellular Health. Curr. Opin. Endocrinol. Diabetes.

[151] Grant W.B., Karras S.N., Bischoff-Ferrari H.A., Annweiler C., Boucher B.J., Juzeniene A., Garland C.F., Holick M.F. (2016). Do Studies Reporting ‘U’-Shaped Serum 25-Hydroxyvitamin D-Health Outcome Relationships Reflect Adverse Effects?. Dermatoendocrinol.

[152] Pilz S., Marz W., Cashman K.D., Kiely M.E., Whiting S.J., Holick M.F., Grant W.B., Pludowski P., Hiligsmann M., Trummer C. (2018). Rationale and Plan for Vitamin D Food Fortification: A Review and Guidance Paper. Front. Endocrinol..

[153] Jaaskelainen T., Itkonen S.T., Lundqvist A., Erkkola M., Koskela T., Lakkala K., Dowling K.G., Hull G.L., Kroger H., Karppinen J. (2017). The Positive Impact of General Vitamin D Food Fortification Policy on Vitamin D Status in a Representative Adult Finnish Population: Evidence from an 11-Y Follow-up Based on Standardized 25-Hydroxyvitamin D Data. Am. J. Clin. Nutr..

[154] Roth D.E., Abrams S.A., Aloia J., Bergeron G., Bourassa M.W., Brown K.H., Calvo M.S., Cashman K.D., Combs G., De-Regil L.M. (2018). Global Prevalence and Disease Burden of Vitamin D Deficiency: A Roadmap for Action in Low- and Middle-Income Countries. Ann. N. Y. Acad. Sci..

[155] Cashman K.D., O’Dea R. (2019). Exploration of Strategic Food Vehicles for Vitamin D Fortification in Low/Lower-Middle Income Countries. J. Steroid Biochem. Mol. Biol..

[156] Aguiar M., Andronis L., Pallan M., Hogler W., Frew E. (2019). The Economic Case for Prevention of Population Vitamin D Deficiency: A Modelling Study Using Data from England and Wales. Eur. J. Clin. Nutr..

[157] Bates B., Lennox A., Prentice A., Bates C., Page P., Nicholson S. (2016). National Diet and Nutrition Survey Results from Years 5 and 6 (Combined) of the Rolling Programme (2012/2013–2013/2014).

[158] O’Neill C.M., Kazantzidis A., Kiely M., Cox L., Meadows S., Goldberg G., Prentice A., Kift R., Webb A.R., Cashman K.D. (2017). A Predictive Model of Serum 25-Hydroxyvitamin D in Uk White as Well as Black and Asian Minority Ethnic Population Groups for Application in Food Fortification Strategy Development Towards Vitamin D Deficiency Prevention. J. Steroid Biochem. Mol. Biol..

[159] Bromage S., Ganmaa D., Rich-Edwards J.W., Rosner B., Bater J., Fawzi W.W. (2018). Projected Effectiveness of Mandatory Industrial Fortification of Wheat Flour, Milk, and Edible Oil with Multiple Micronutrients among Mongolian Adults. PLoS ONE.

[160] Bromage S., Gonchigsumlaa E., Traeger M., Magsar B., Wang Q., Bater J., Li H., Ganmaa D. (2019). Awareness and Attitudes Regarding Industrial Food Fortification in Mongolia and Harbin. Nutrients.

